# Supplementary material for: The genome sequence of the cylindrical bark beetle, Colydium elongatum (Fabricius, 1787) (Coleoptera: Zopheridae)
Source: Wellcome Open Res. 2025 Nov 5;10:623. [Version 1] doi: 10.12688/wellcomeopenres.25078.1 (PMC12770892; doi:10.12688/wellcomeopenres.25078.1)
Supplement: Peer review history

# Reviewer report: Reviewer response for version 1

**DOI:** 10.21956/wellcomeopenres.27645.r141907

**Published:** 2026-01-20

**Authors:** Arun Arumugaperumal

**Affiliations:** 1 Department of Biotechnology, Rajalakshmi Engineering College, Chennai, Tamil Nadu, India

The genome note describes the genome sequencing of the cylindrical bark beetle *Colydium elongatum*. The authors have used a male specimen for the sequencing project. They have used PacBio long-read sequencing and Hi-C sequencing data to arrive at an assembly of size 450.16 Mb. Since a male had been used for sequencing, they have obtained the X and Y chromosomes. The mitogenome has also been assembled. They have identified a total of 13,883 protein-coding genes in the assembly. The quality of the assembly is evident from the contig N50 value of 2.41 Mb. BUSCO analysis has also shown a completeness of 99.7% with respect to the endopterygota_odb10 dataset.

 The QV value has been estimated as 60.8, but the EBP metric mentioned is 6.C.Q63. The grant information has an url which appears as text itself.

Are sufficient details of methods and materials provided to allow replication by others?

Yes

Is the rationale for creating the dataset(s) clearly described?

Yes

Are the datasets clearly presented in a useable and accessible format?

Yes

Are the protocols appropriate and is the work technically sound?

Yes

Reviewer Expertise:

Bioinformatics; Genomics

I confirm that I have read this submission and believe that I have an appropriate level of expertise to confirm that it is of an acceptable scientific standard.

# Reviewer report: Reviewer response for version 1

**DOI:** 10.21956/wellcomeopenres.27645.r141904

**Published:** 2026-01-08

**Authors:** Chenyang Cai

**Affiliations:** 1 Chinese Academy of Sciences, Beijing, Beijing, China

This manuscript presents a high-quality, chromosome-level genome assembly for *Colydium elongatum*, representing the first genomic resource for the genus *Colydium* and one of the few assemblies available for the zopherid subfamily Colydiinae. The study follows the standard Darwin Tree of Life pipeline and provides extensive methodological detail, strong assembly metrics, and a well-documented workflow from sampling to quality assessment. The dataset will clearly be a valuable reference for coleopteran comparative genomics, systematics, and studies of saproxylic insects. the manuscript is clearly written, well structured, and methodologically sound. As this represents the first genomic resource for the genus, it would strengthen the manuscript to expand briefly on: 1) The phylogenetic position of Zopheridae within Tenebrionoidea and 2) What evolutionary questions this genome may help address (e.g., adaptations to saproxylic habitats, body elongation, feeding strategies).

Are sufficient details of methods and materials provided to allow replication by others?

Yes

Is the rationale for creating the dataset(s) clearly described?

Yes

Are the datasets clearly presented in a useable and accessible format?

Yes

Are the protocols appropriate and is the work technically sound?

Yes

Reviewer Expertise:

beetles; phylogenomics; genomes

I confirm that I have read this submission and believe that I have an appropriate level of expertise to confirm that it is of an acceptable scientific standard.

# Reviewer report: Reviewer response for version 1

**DOI:** 10.21956/wellcomeopenres.27645.r141909

**Published:** 2026-01-05

**Authors:** Lindsey C Perkin

**Affiliations:** 1 SPARC, ICCDRU, United States Department of Agriculture ARS, College Station, TX, USA

This report is well written. I only have a couple comments.

 rationale: while I agree everything should be sequenced without question, it would be nice to learn why it is important to sequence C. elongatum. Pest? Important to the ecosystem? This was partially described but more would be nice.

 methods: was the second specimen used for Hi-C also male?

Are sufficient details of methods and materials provided to allow replication by others?

Yes

Is the rationale for creating the dataset(s) clearly described?

Partly

Are the datasets clearly presented in a useable and accessible format?

Yes

Are the protocols appropriate and is the work technically sound?

Yes

Reviewer Expertise:

weevil genomics

I confirm that I have read this submission and believe that I have an appropriate level of expertise to confirm that it is of an acceptable scientific standard.

# Reviewer report: Reviewer response for version 1

**DOI:** 10.21956/wellcomeopenres.27645.r138748

**Published:** 2025-11-21

**Authors:** Zhonghua Wei

**Affiliations:** 1 China West Normal University, Nanchong, China

This study presents the genome assembly and annotation of Colydium elongatum, which holds scientific significance. However, some questions need to be addressed before it can be accepted for indexing.

 1. Figure 1 was blurry, please provide a clear image for this species.

 2. The genome annotation file should be publicly accessible to allow for the verification of potential issues in the annotation.

 3. In the References section, some journals are not abbreviated, such as: Deutsche Entomologische Zeitschrift and Annales Zoologici. Please do the needful.

Are sufficient details of methods and materials provided to allow replication by others?

Yes

Is the rationale for creating the dataset(s) clearly described?

Yes

Are the datasets clearly presented in a useable and accessible format?

Yes

Are the protocols appropriate and is the work technically sound?

Yes

Reviewer Expertise:

NA

I confirm that I have read this submission and believe that I have an appropriate level of expertise to confirm that it is of an acceptable scientific standard.
